# Influenza a Neuraminidase-Based Bivalent mRNA Vaccine Induces Th1-Type Immune Response and Provides Protective Effects in Mice

**DOI:** 10.3390/vaccines12030300

**Published:** 2024-03-13

**Authors:** Mingyang Li, Mengyuan Liu, Shaohui Song, Ruirui Zhao, Yun Xie, Jing Liu, Lilan Xu, Xuefeng Ma, Mingyu Song, Jian Zhou, Guoyang Liao

**Affiliations:** Institute of Medical Biology, Chinese Academy of Medical Sciences & Peking Union Medical College, Kunming 650118, China; limingyang0917@student.pumc.edu.cn (M.L.); liumengyuan@student.pumc.edu.cn (M.L.);

**Keywords:** mRNA vaccine, influenza A, neuraminidase, Th1

## Abstract

Vaccines are one of the most effective means of preventing influenza A, typically containing the hemagglutinin (HA) of the influenza A virus. However, antigenic drift and shift of the influenza A virus can lead to instability in vaccine efficacy. Compared to HA, the antigenic variation rate of neuraminidase (NA) is slower. In traditional inactivated influenza vaccines, although they contain a certain amount of NA, there are significant differences between different batches, which cannot consistently induce NA-based immune responses. Therefore, NA is often overlooked in vaccine development. In this study, we report an mRNA vaccine encoding the NA of two strains of influenza A virus. The experimental results demonstrated that when matched with the viral strain, this mRNA vaccine induced high levels of neutralizing antibodies, providing a protective effect to mice in viral challenge experiments, and this immune response was shown to be biased towards the Th1 type. In summary, this study demonstrates that NA is a promising potential antigen, providing new insights for the development of influenza A virus vaccines.

## 1. Introduction

Influenza A virus (IAV) can be classified into different subtypes based on the antigenicity of its hemagglutinin (HA) and neuraminidase (NA), with 18 HA subtypes and 11 NA subtypes known [1].

Vaccination is one of the most effective ways to prevent influenza, and currently, there are four main types of influenza vaccines available: egg-based inactivated split vaccines [2], cell-culture-based inactivated vaccines [3], recombinant subunit vaccines [4], and virus vector-based attenuated vaccines [5]. The major antigen of these vaccines is the HA protein of the influenza virus, which induces neutralizing antibodies specific to the targeted influenza virus subtype, preventing viral attachment to host cells and thereby inhibiting viral infection and replication. However, existing influenza vaccines have several limitations. Firstly, their efficacy is unstable due to antigenic variations in the influenza virus, leading to potential mismatches between the HA antigen in the vaccine and circulating viruses, resulting in reduced or ineffective vaccine efficacy [6]. Secondly, the production cycle is lengthy as influenza vaccine production relies on complex biological processes such as egg-based or cell-culture-based methods. This typically requires a production cycle of more than six months, limiting the supply and distribution of influenza vaccines due to time and resource constraints. Lastly, influenza vaccines have limited suitability for certain population groups. The immunogenicity and safety of influenza vaccines may vary among different populations, such as the elderly, children, pregnant women, and immunocompromised individuals [7,8], necessitating evaluation and adjustments of vaccine efficacy and safety across diverse population groups.

Another antigenic target option is the NA protein, a tetrameric transmembrane protein with a hydrophobic sequence at the amino terminus that possesses enzymatic activity to cleave terminal sialic acids from glycoproteins on the surface of host cells [9]. Compared to HA, NA exhibits slower antigenic variation rates [10,11]. However, NA has been largely overlooked in vaccine development, and although a certain amount of NA is present in traditional influenza inactivated vaccines, there are significant differences between batches, resulting in an inability to consistently induce NA-based immune responses [12,13,14].

Currently, multiple studies have shown the effectiveness of mRNA vaccines for influenza. The first influenza mRNA vaccine to enter clinical trials encodes a modified-length membrane-bound HA of highly pathogenic avian influenza strains H10N8 (A/Jiangxi-Donghu/346/2013) and H7N9 (A/Anhui/1/2013), which induce robust humoral immunity in subjects after two doses [15]. Currently, some influenza mRNA vaccine candidates, mRNA1010 [16], pF-07252220 [17], CVSQIV [18], are in clinical trials, all of which have HA antigens selected. However, in this study, we designed an mRNA vaccine encoding two types of influenza A virus NAs to demonstrate the potential antigen protection of NA. This provides new insights into the design and improvement of influenza A virus vaccines to improve vaccine efficacy.

## 2. Materials and Methods

### 2.1. Virus Strains

The A/Michigan/45/2015 (16/248 E13) strain of H1N1 influenza virus and the A/Hong Kong/4801/2014 (15/184 E2) strain of H3N2 influenza virus were used in this study. After purification, these strains were stored at −80 °C. The half-maximal tissue culture infectious dose (TCID_50_) of A/Michigan/45/2015 was 10^−6.29^, and the half-maximal lethal dose (LD_50_) was 10^−2.32^. The TCID_50_ of A/Hong Kong/4801/2014 was 10^−5.52^, and the LD_50_ was 10^−2.25^.

### 2.2. mRNA Production

The three sequences, including P2A [19], A/Michigan/45/2015 (H1N1)-NA (N1, EPI841264), and A/Hong Kong/4801/2014 (H3N2)-NA (N2, EPI73147) sequences were codon-optimized, synthesized (GenScript Co., Ltd., Nanjing, China), and cloned into mRNA production plasmids. Influenza A mRNA was produced using T7 RNA polymerase (Vazyme Co., Ltd., Nanjing, China), CleanCap (Syngenebio Co., Ltd., Nanjing, China), and a mixture of triphosphates (with m1Ψ-5′-triphosphate replacing UTP, Syngenebio Co., Ltd., Nanjing, China). In vitro transcription was performed according to the protocol provided by the manufacturer (Vazyme Co., Ltd., Nanjing, China).

### 2.3. LNP Package

The Influenza A N1 + N2 mRNA was encapsulated in LNPs, which consisted of a mixture of ionizable cationic lipids, phosphatidylcholine, cholesterol, and polyethylene glycol lipids, with a molar ratio of 46.3:9.4:42.7:1.6 [20]. This mixture was rapidly mixed with an aqueous solution containing the mRNA at acidic pH. The LNPs were stored at −80 °C at a concentration of 300 μg/mL. The average particle size of these Influenza A N1 + N2 mRNA vaccines was 90 nm, with a polydispersity index of <0.05, and an encapsulation efficiency of >95%.

### 2.4. Mouse Immunization Program

The specific pathogen-free BALB/c mice, 6 weeks old, female, weighing 14–17 g, were provided by the Institute of Medical Biology, Chinese Academy of Medical Sciences, and the Center Service Department of Peking Union Medical College (IMB, CAMS). These mice were randomly divided into the experimental group and the control group, with 5 mice in each group (n = 5). In the experimental group, each mouse was intramuscularly injected with 30 μg of Influenza A N1 + N2 mRNA vaccine in the hind limb muscles, while in the control group, mice were injected with an equal volume of Tris solution (25 mM) using the same method. All experiments were conducted in accordance with the principles of the IMB Animal Ethics Committee’s “Guidelines for the Care and Use of Laboratory Animals” (license number: SCXK (dian) K2022-0002).

### 2.5. Mouse Serum Isolation

The mice were anesthetized using ketamine, and blood samples were collected via cardiac puncture. The collected blood was allowed to clot overnight at 4 °C, and then centrifuged at 3000 rpm for 10 min to collect the serum.

### 2.6. Microneutralization Test

MDCK cells were seeded at a density of 2.5 × 10^4^ cells per well in a 96-well culture plate and incubated overnight at 37 °C with 5% CO_2_. Following the instructions, serum samples were treated with receptor destroying enzyme (RDE) (Seiken Co., Ltd., Tokyo, Japan) working dilution for 16 h at 37 °C in a 4:1 ratio. After 16 h, the serum samples were incubated at 56 °C for 30 min to inactivate the RDE. Diluted serum samples and the virus were mixed using virus maintenance medium (containing 2 μg/mL TPCK-trypsin and 10% BSA in DMEM). The previously treated serum samples were first diluted to 1:20 and then serially diluted in a 2-fold dilution series, with a final dilution of 1:1024. The virus was diluted to 200× TCID_50_/100 μL. The diluted serum and virus were mixed in equal volumes, and incubated at 37 °C for 1 h. The MDCK cells were washed with PBS and then 100 μL of the serum/virus mixture was added to the cells, followed by incubation at 34 °C with 5% CO_2_ for 72 h. Afterward, 50 μL of cell supernatant was mixed with 50 μL 1% chicken red blood cells for hemagglutination assay to assess the neutralizing potential of the serum.

### 2.7. Virus Challenge Experiments

The mice were randomly divided into experimental and control groups, with 5 mice in each group (n = 5). In the experimental group, each mouse was intramuscularly injected with 30 μg of Influenza A N1 + N2 mRNA vaccine in the hind limb muscles, while in the control group, mice were injected with an equal volume of Tris solution (25 mM), following the same procedure as the experimental group. On day 21, the mice were subjected to a viral challenge experiment. The initial body weight of each mouse was measured. After anesthetizing the mice with chloral hydrate, 10 μL of virus suspension was drawn up using a micropipette and administered as drops into both nostrils of the mice. Each mouse received a total of 100× LD_50_ virus dosage. Starting from day 22, the mice were monitored for changes in body weight for 14 consecutive days. If the mouse’s body weight was ≤80% of the corresponding initial body weight, it was recorded as dead.

### 2.8. Mouse Spleen Lymphocyte Isolation

A total of 5 mL of 1× mouse lymphocyte separation medium was added to a 6-well cell culture plate and placed in a cell strainer. The mouse spleen was placed on the cell strainer and ground with the inner core of a syringe fitted with a rubber head. The spleen cells were passed through the cell strainer and transferred to a 15 mL centrifuge tube, with 1 mL of RPMI 1640 medium added slowly. The tube was centrifuged at 300× *g* for 5 min, and the supernatant was discarded. Then, 10 mL of red blood cell lysis solution was added to the centrifuge tube, and the mixture was incubated at room temperature for 5 min. Afterward, 40 mL of PBS was added to the centrifuge tube and centrifuged at 300× *g* for 5 min. The supernatant was discarded, and 700 μL of FACS buffer was added to resuspend the cells, creating a spleen lymphocyte suspension.

### 2.9. ELISA Assay Specific IgG Antibody Types and Titers

The antigen (N1: Influenza A H1N1 (A/Michigan/45/2015) Neuraminidase; N2: Influenza A H3N2 (A/Hong Kong/4801/2014) Neuraminidase, purchased from SinoBiological, Beijing, China) was diluted to a concentration of 2 μg/mL in 0.05 M, pH 9.4 carbonate buffer, and added to a 96-well plate at 100 μL per well. The plate was coated at 4 °C for 16 h. The plate was washed three times with PBST (0.01 M PBS containing 0.05% (*v*/*v*) Tween-20) and then blocked with 200 μL of 10% (*v*/*v*) BSA (prepared in 0.01 M PBS) at 37 °C for 2 h. After blocking, the plate was washed three times with PBST and incubated with serially diluted mouse serum at 37 °C for 2 h. Next, the plate was washed three times with PBST and incubated with anti-mouse IgG1/IgG2a HRP conjugated secondary antibody (ThermoFisher Scientific, Beijing, China) at 37 °C for 1 h. The plate was washed five times with PBST and TMB substrate (BD Bioscience, Shanghai, China) was added. The plate was incubated at room temperature in the dark for 10 min, and the reaction was stopped with 2 M sulfuric acid. The absorbance (OD_450_) was measured using a microplate reader (Bio-Tek, Beijing, China). A sample with an OD_450_ greater than 2.1 times the OD_450_ value of the negative serum was considered positive, and the antibody titer was determined as the highest serum dilution that produced a positive result.

### 2.10. Th1/Th2 Cells Were Detected by Flow Cytometry

Dilute the following antibodies in Brilliant Stain Buffer Plus (BD Pharmingen, Shanghai, China): CD3 1:50–100, CD4 1:400, CD8 1:200, CD30 1:100, CD195 1:100, IL-4R 1:50 (BD Pharmingen), IL12R 1:10 (R&D SYSTEM, Shanghai, China). Mix well. Take 6.5 × 10^6^–1.0 × 10^7^ splenic lymphocytes and add it to a “U” bottom 96-well plate (Corning, Suzhou, China). Add the antibody mixture and incubate at 4 °C in the dark for 30 min. Add 100 μL of FACS Buffer to each well, mix well, and centrifuge at 300× *g* for 5 min. Discard the supernatant. Add 200 μL of FACS Buffer to each well, mix well, and centrifuge at 300× *g* for 5 min. Discard the supernatant. Resuspend the cells in 200 μL of PBS in each well. Add 10 μL of 7-AAD (4A Biotech Co., Ltd., Suzhou, China), mix well, and proceed with flow cytometry analysis (Attune NxT, ThermoFisher Scientific). We entrusted 4A BIOTECH to test the samples and to perform the data analysis.

### 2.11. Elispot

Following the manufacturer’s guidelines for Elispot, wash the plate 5 times with PBS (10 mM). Add 100 μL of RPMI 1640 medium (containing 10% FBS) to each well, and incubate at room temperature for 30 min. In each well of the IL-4 plate, seed 2.5 × 10^4^ mouse splenic lymphocytes (Mabtech, Nacka Strand, Sweden). In each well of the IFN-γ plate, seed 5 × 10^4^ mouse splenic lymphocytes (Mabtech, Nacka Strand, Sweden). Add the antigen-stimulating substances (N1 or N2, as described in Section 2.9, purchased from SinoBiological, Beijing, China) in experimental wells, add 10 μL of PMA+Lonomycin mixture (Dakewe Biotech Co., Ltd., Shenzhen, China) to the positive control wells, while the negative control wells contain cells and RIPA 1640 medium only. Incubate the plate at 37 °C in a 5% CO_2_ incubator for 36 to 48 h. Discard the cell suspension from the wells and wash the plate 4 times with PBS. Dilute the detection antibodies BVD6-24G2-biotin and R4-6A2-biotin in PBS containing 0.5% FBS at a 1:1000 ratio. Add 100 μL of diluted antibodies to each well and incubate at room temperature for 2 h. Discard the liquid from the wells and wash the plate 4 times with PBS. Dilute Streptavidin-ALP in PBS containing 0.5% FBS at a 1:1000 ratio. Add 100 μL of diluted Streptavidin-ALP to each well and incubate at room temperature for 1 h. Discard the cell suspension from the wells and wash the plate 5 times with PBS. Filter the substrate solution using a 0.45 μm needle filter and add 100 μL to each well. Incubate at room temperature in the dark for 15 min. Rinse the plate slowly with running water to stop the color development once distinct spots appear in the wells. Leave the plate to dry completely at room temperature in the dark overnight. Finally, use an Elispot reader for plate analysis (CTL, Shaker Heights, OH, USA).

### 2.12. Statistics

Perform statistical analysis of the experimental data using the R programming environment (version 4.2.1) with the Stats Package (version 4.2.1) and Car Package (version 3.1). A significance level of * *p* < 0.05, ** *p* < 0.01, and *** *p* < 0.001 is considered to indicate significant differences in the statistical results.

## 3. Results

### 3.1. Influenza A N1 + N2 mRNA Vaccine Design

**Figure 1:**
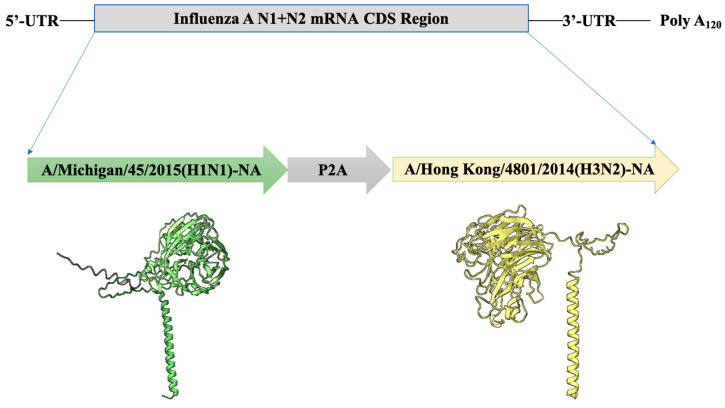
Influenza A N1 + N2 mRNA vaccine design. N1: A/Michigan/45/2015 (H1N1)-NA (green); N2: A/Hong Kong/4801/2014 (H3N2)-NA (yellow).

We used the Porcine Teschovirus-1 2A sequence (P2A) to link the A/Michigan/45/2015 (H1N1)-NA and A/Hong Kong/4801/2014 (H3N2)-NA sequences on the same mRNA molecule. During the translation of the Influenza A N1 + N2 mRNA, the ribosome undergoes a “self-cleavage” between the glycine and proline residues at the C-terminus of the P2A sequence, resulting in the independent formation of the A/Michigan/45/2015 (H1N1)-NA and A/Hong Kong/4801/2014 (H3N2)-NA proteins (Figure 1).

### 3.2. Influenza A N1 + N2 mRNA Induces the Production of Specific Neutralizing Antibodies

**Figure 2:**
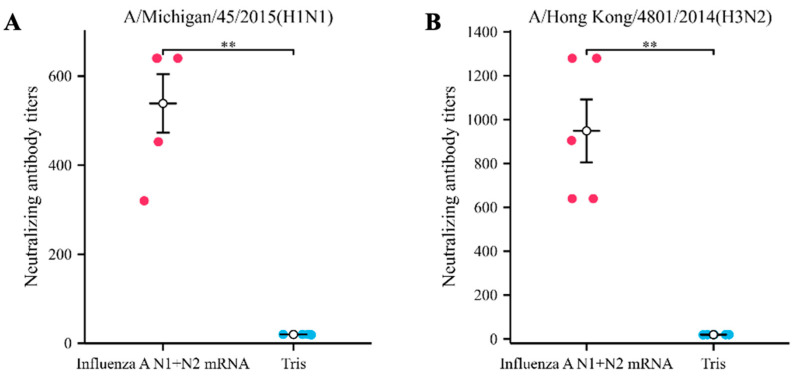
Induction of specific neutralizing antibodies by the Influenza A N1 + N2 mRNA vaccine. (**A**). Neutralizing activity of mouse sera from Influenza A N1 + N2 mRNA-immunized mice against A/Michigan/45/2015 (H1N1). (**B**). Neutralizing activity of mouse sera from Influenza A N1 + N2 mRNA-immunized mice against A/Hong Kong/4801/2014 (H3N2). ** *p* < 0.01.

To evaluate the ability of the Influenza A N1 + N2 mRNA vaccine to induce antibody responses, we immunized mice twice on day 0 and day 14. The experimental group received intramuscular injections of 30 μg of Influenza A N1 + N2 mRNA vaccine in the hind limbs, while the control group mice were injected with an equal volume of Tris solution. On day 21, we collected mouse sera for virus neutralization assays and found that the Influenza A N1 + N2 mRNA vaccine induced the production of neutralizing antibodies against both the A/Michigan/45/2015 (H1N1) and A/Hong Kong/4801/2014 (H3N2) strains in mice. The antibody titers were high, with a maximum titer of 1:1024 (Figure 2). Similar neutralizing effects were observed in the sera of the Tris group mice; however, this effect disappeared at dilutions higher than 1:20, which can be considered the “background value”.

### 3.3. Influenza A N1 + N2 mRNA Provides Protection in Mice in Viral Challenge Experiments

**Figure 3:**
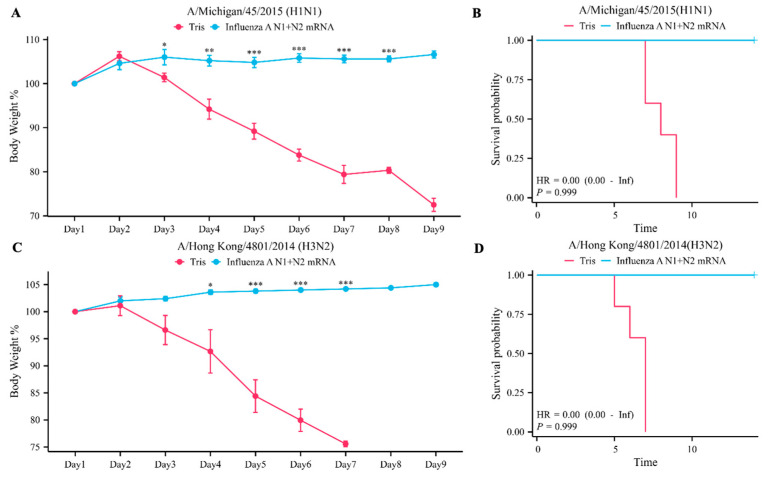
Protective effect of the Influenza A N1 + N2 mRNA vaccine in viral challenge experiments. (**A**). Statistical analysis of mouse body weight changes in the A/Michigan/45/2015 (H1N1) viral cha −lenge experiment. (**B**). KM curve analysis of mouse survival in the A/Michigan/45/2015 (H1N1) viral challenge experiment. (**C**). Statistical analysis of mouse body weight changes in the A/Hong Kong/4801/2014 (H3N2) viral challenge experiment. (**D**). KM curve analysis of mouse survival in the A/Hong Kong/4801/2014 (H3N2) viral challenge experiment. * *p* < 0.05, ** *p* < 0.01, and *** *p* < 0.001.

To assess the protective efficacy of the Influenza A N1 + N2 mRNA vaccine against viral challenge in mice, as described earlier, we immunized mice twice on day 0 and day 14 and subjected them to intranasal challenge on day 21. The results of the experiment showed that in the Tris group, all mice exhibited significant weight loss after intranasal challenge with 100× median lethal dose (LD_50_) of the influenza A virus, whether it was A/Michigan/45/2015 (H1N1) or A/Hong Kong/4801/2014 (H3N2) (Figure 3A,C). In contrast, the mice in the experimental group remained alive after continuous observation for 14 days (Figure 3B,D). This indicates that the Influenza A N1 + N2 mRNA vaccine provides strong protection against viral challenge in mice, preventing them from succumbing to the virus. However, it is important to note that this protection is contingent upon matching viral strains.

### 3.4. Influenza A N1 + N2 mRNA Vaccine Induces Specific T Cell Responses

**Figure 4:**
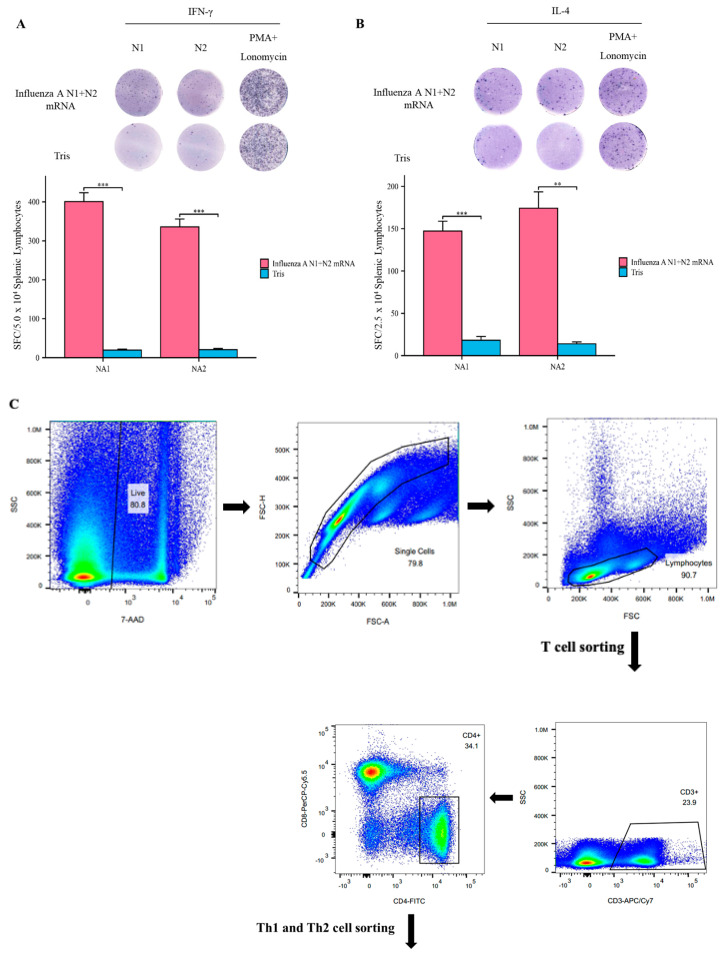

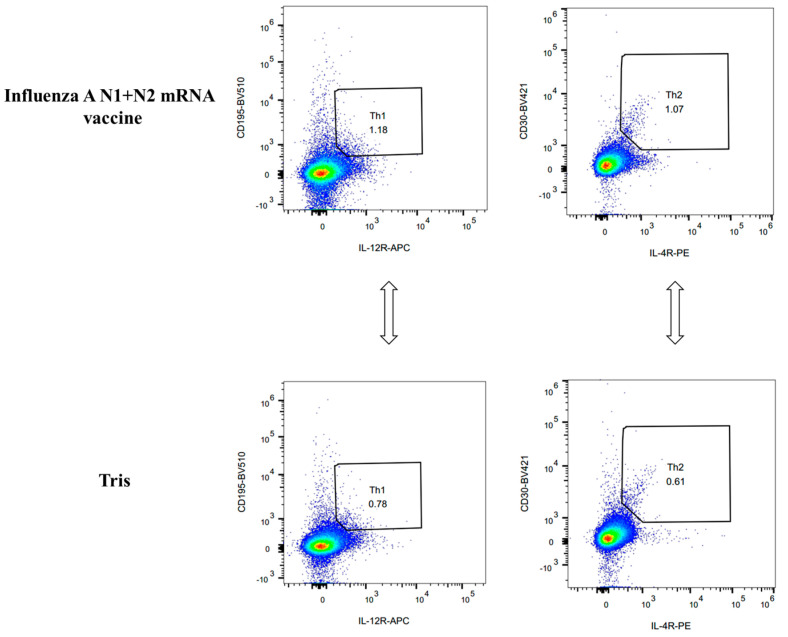
Influenza A N1 + N2 mRNA vaccine stimulates the production of different types of cytkines by splenic lymphocytes. (**A**). IFN−γ: Elispot assay (**top**), spot count bar graph (**bottom**). (**B**). IL−4: Elispot assay (**top**), spot count bar graph (**bottom**). (**C**). Flow cytometry analysis of the ratio of Th1 and Th2 cells. ** *p* < 0.01, and *** *p* < 0.001.

To further confirm the specificity of the T cell response induced by the Influenza A N1 + N2 mRNA vaccine, we stimulated splenic lymphocytes from mice at day 21 with specific antigens and detected the secretion of cytokines using Elispot. Upon stimulation with the specific antigens NA1 and NA2, the experimental group mice showed significantly higher levels of IFN-γ and IL-4 secretion by splenic lymphocytes compared to the Tris group mice (Figure 4A,B). The flow cytometry analysis also revealed the proliferation of helper T cells (Figure 4C). These findings indicate that the Influenza A N1 + N2 mRNA vaccine can induce a specific T cell response.

### 3.5. The Influenza A N1 + N2 mRNA Vaccine Stimulates the Production of Different Types of IgG Antibodies

**Figure 5:**
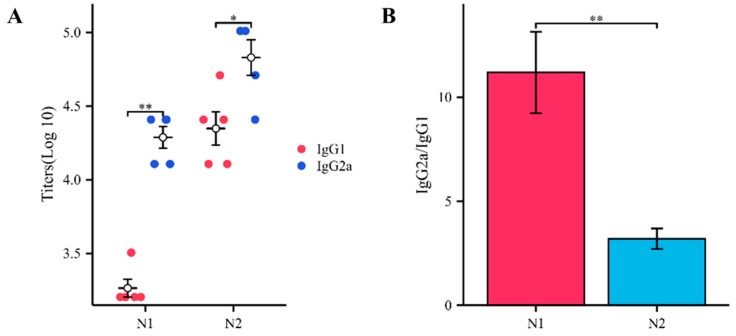
Influenza A N1 + N2 mRNA vaccine stimulates the production of different types of IgG antibodies. (**A**). ELISA assay determining the titers of IgG2a and IgG1 antibodies against NA in the serum of immunized mice. (**B**). Ratio of IgG2a to IgG1 antibodies against NA in the serum of immunized mice. * *p* < 0.05, and ** *p* < 0.01.

To evaluate the Th1–Th2 balance, we used ELISA to detect specific anti-NA (NA1 and NA2) antibodies in the serum of the experimental group mice. The ELISA results showed that the titer of IgG2a antibodies in the serum of the experimental group mice was significantly higher than that of the IgG1 antibodies. Similar results were observed in both the NA1 and NA2 groups, consistent with previous findings, indicating that the immune response induced by the Influenza A N1 + N2 mRNA vaccine in mice is more biased towards the Th1 type (Figure 5A). Interestingly, theoretically, the two antigens A/Michigan/45/2015 (H1N1)-NA and A/Hong Kong/4801/2014 (H3N2)-NA, connected by the P2A sequence, should be consistent at the protein level. However, from the experimental results, the titer of anti-NA2 antibodies was higher than that of NA1, regardless of IgG1 or IgG2a, which may be related to the characteristics of the viral antigen itself. However, when calculating the ratio of IgG2a/IgG1, this phenomenon was reversed, suggesting that NA1 may stimulate a higher degree of Th1 bias in mice compared to NA2 (Figure 5B).

## 4. Discussion

Although vaccine production technologies for seasonal influenza viruses are well-established, traditional influenza vaccines have predominantly targeted the HA antigen. However, the development of novel vaccine platforms has focused on highly conserved antigens such as M2e [21], HA stem region [22,23,24], NP [25], and M1 [26], with the aim of inducing robust cross-protective antibody responses and cross-reactive T cell responses. The antigenicity of NA, on the other hand, has often been overlooked. Currently, a series of antibodies targeting the active sites of NA have been identified, which have been isolated from individuals who have been infected with influenza or have been vaccinated, and have shown broad protective effects. Moreover, it is important to note that the antigenicity of NA is not closely related to HA. Therefore, even when there is antigenic drift in HA, the existing NA-specific antibodies can still prevent the replication of the new virus [27]. These pieces of evidence indicate that NA possesses good immunogenicity and can be incorporated into the design of influenza vaccines to enhance their efficacy.

In this study, we designed an mRNA vaccine capable of simultaneously encoding two NA proteins from the A/Michigan/45/2015 (H1N1) and A/Hong Kong/4801/2014 (H3N2) virus strains. These two NA proteins were separated by a P2A sequence. Theoretically, when the lipid nanoparticle (LNP) carrying the Influenza A N1 + N2 mRNA enters host cells and undergoes successful translation, the ribosome would undergo “self-cleavage” between the glycine and proline residues at the C-terminus of the P2A sequence [28], resulting in the separation of the two NA proteins and their independent folding into individual proteins. However, due to the lack of suitable antibodies for detecting these two NA proteins, there is no direct evidence to suggest that these two NA proteins can form homologous or heterologous tetramers in vivo, as designed.

Traditional vaccines typically consist of specific antigens and adjuvants, which help stimulate adaptive immune responses, while adjuvants can stimulate innate immune responses and activate T cells [29]. In mRNA vaccines, the mRNA molecule itself has immunostimulatory properties, so it plays a dual role as both an antigen and an adjuvant. After intramuscular injection, the adaptive immune system can be activated through several pathways: first, uptake by non-APCs such as muscle cells and epithelial cells; second, uptake by tissue-resident immune cells such as dendritic cells (DCs) and macrophages, which in turn activate T and B cells, triggering an immune response [30]; third, limited diffusion into lymph nodes or organs such as the liver, lungs, and spleen [31]. Previous studies have shown that mRNA vaccines encoding HA prepared using modified nucleotides can induce robust Tfh cell responses and germinal center formation, leading to the production of long-lasting and high-affinity antibodies [32,33]. In our study, we used N1-Me-Pseudo-UTP (M1Ψ) to prepare the Influenza A N1 + N2 mRNA vaccine. After two injections into the hind limbs of immunized mice, high titers of NA-specific antibodies were detected in the serum, including neutralizing antibodies. The highest neutralizing antibody titer against A/Michigan/45/2015 (H1N1) was 1:640, with an average geometric titer of 519.8. The highest neutralizing antibody titer against A/Hong Kong/4801/2014 (H3N2) was 1:1024, with an average geometric titer of 905.1. These results indicate that NA, like HA, has good immunogenicity based on mRNA vaccine technology, and can induce high levels of neutralizing antibodies against influenza A viruses. Additionally, experiments have suggested that B cell responses to NA may occur more rapidly than those to HA [34]. Furthermore, we demonstrated through viral challenge experiments that the Influenza A N1 + N2 mRNA vaccine provided protection to mice against both H1N1 and H3N2 strains at 100-fold LD_50_ conditions, with a 100% survival rate and no significant weight loss. It has also been reported that antibodies targeting NA have been identified to cross-react with both IAV and IBV [35]. However, due to the limited number of strains tested, we are currently unable to assess cross-reactivity with other influenza viruses.

The T helper (Th) cell hypothesis was first proposed by Mosmann et al. and is mainly divided into the Th1 and Th2 subsets [36]. Th1 and Th2 cells differentiate from the initial CD4+ T cells (Th0) through stimulation by different signaling molecules to coordinate various adaptive immune responses. Th1 cells secrete cytokines such as IFN-γ, which play a role in cellular immunity [37]. On the other hand, Th2 cells primarily secrete cytokines such as IL-4, which play a role in humoral immunity by stimulating B cell proliferation and antibody production [38]. Traditional vaccines, such as inactivated or split vaccines, usually have poor immunogenicity and require the addition of adjuvants such as alum to stimulate sufficient immune responses, with alum primarily inducing Th2 responses [39]. After vaccination with mRNA vaccines, the target antigen is translated from the host cell cytoplasm. The target antigen is then degraded in the proteasome to generate antigenic epitopes. These epitopes form complexes with MHC class I molecules and are exposed on the cell surface, mimicking the natural process of viral infection. Moreover, the intrinsic properties of mRNA can induce the production of type I interferon (IFN) and stimulate the differentiation of Th0 cells into Th1 cells, thereby triggering a Th1-type immune response [40]. In our study, after two intramuscular injections of the Influenza A N1 + N2 mRNA vaccine, both Th1 and Th2 cells showed significant proliferation in the spleen of the immunized mice (Th1: 1.18 vs. 0.78; Th2: 1.07 vs. 0.61). The Elispot results further demonstrated that the proliferation of the Th1 and Th2 cells was antigen-specific, meaning that both the NA1 and NA2 antigens could stimulate the secretion of IFN-γ and IL-4 by lymphocytes in the spleen of immunized mice. Additionally, to evaluate the Th1–Th2 balance, we used ELISA to measure the titers and ratios of the IgG2a antibodies (Th1 type) and IgG1 antibodies (Th2 type) in the serum of the immunized mice. The results showed that the titer of the IgG2a antibodies was significantly higher than that of the IgG1 antibodies, and the IgG2a/IgG1 ratio was >1, indicating that the LNP-IAV NA1+NA2 mRNA vaccine induced a Th1-biased immune response in mice, and NA1 seemed to be more able to induce this phenomenon.

## 5. Conclusions

mRNA vaccines are a novel vaccine technology that, upon entering cells, can efficiently translate nucleic acid sequences into antigen proteins through the ribosome, stimulating the body to generate an immune response. They have several advantages, including a high antigen expression efficiency, strong immunogenicity, and the ability to rapidly respond to antigen variations.

In our study, we successfully prepared an mRNA vaccine encoding the neuraminidase protein of influenza A virus, demonstrating that the “N1-P2A-N2” mRNA vaccine has good immune protective properties. This indicates that NA is an immunogenic potential antigen, offering new insights for the development of influenza virus vaccines.

## Data Availability

The data used to support the findings of this study are included within the article.
